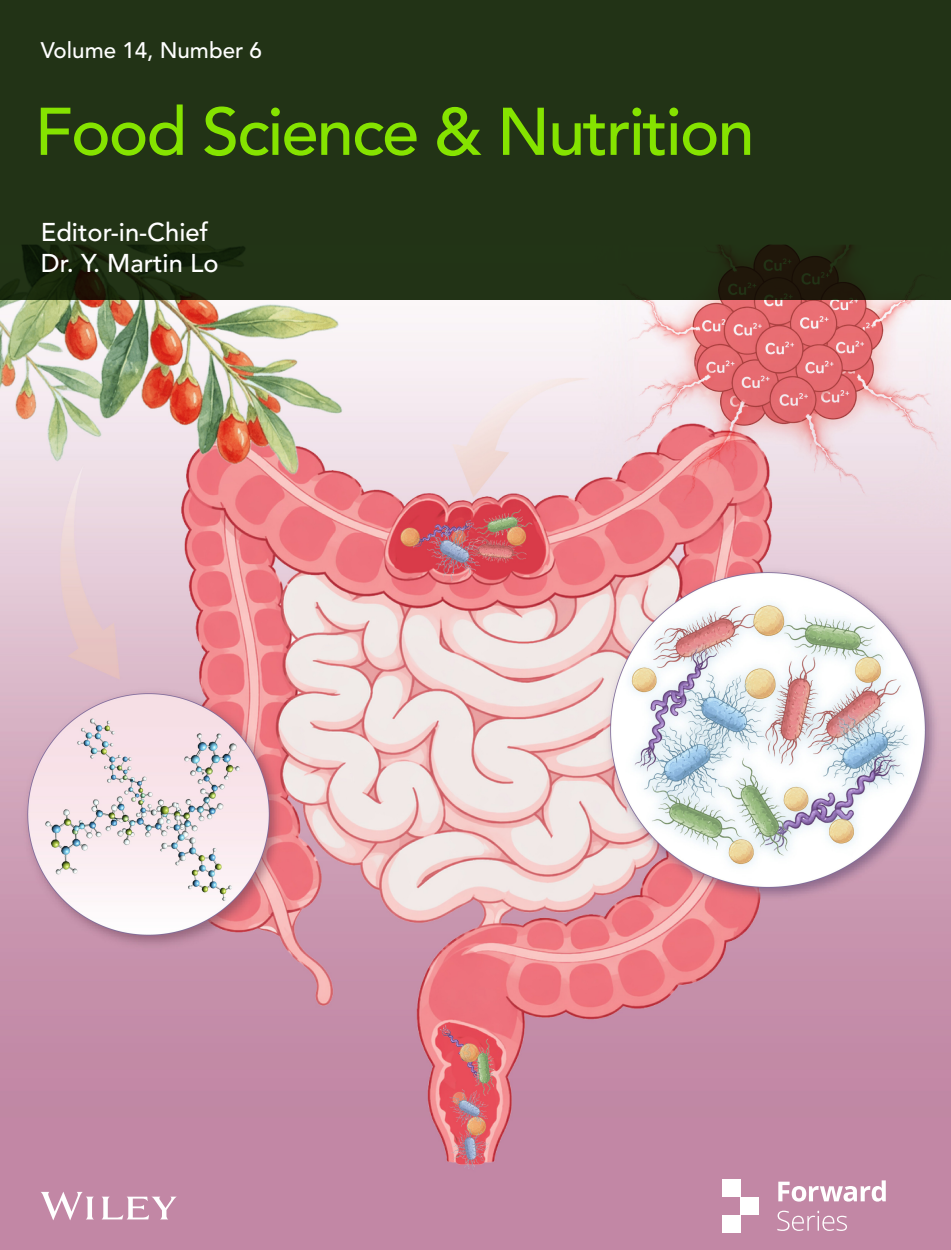# Cover Image

**DOI:** 10.1002/fsn3.71817

**Published:** 2026-06-26

**Authors:** Shuzhen Fang, Kangyi Zhang, Xiang Fang, Danqing Liu, Yulong Yang, Guijie Chen, Wenming Yang

## Abstract

The cover image is based on the article *Prebiotic Effects of Lycium barbarum Polysaccharides on Gut Microbiota and Short‐Chain Fatty Acids Production in Wilson's Disease: An In Vitro Fermentation Study* by Shuzhen Fang *et al.,* https://doi.org/10.1002/fsn3.71815.